# Effectiveness and safety of acupuncture for chronic obstructive pulmonary disease: A protocol for systematic review and meta-analysis

**DOI:** 10.1097/MD.0000000000028555

**Published:** 2022-01-14

**Authors:** Tian-Yi You, Hao-Yang Zhang, Ji-Quan Li, Tie-Ming Ma

**Affiliations:** a Graduate School, Liaoning University of Traditional Chinese Medicine, Shenyang, Liaoning, China; b College of acupuncture and massage, Liaoning University of Traditional Chinese Medicine, Shenyang, Liaoning, China.

**Keywords:** acupuncture, chronic obstructive pulmonary disease, meta-analysis, systematic review

## Abstract

**Background:**

Chronic Obstructive Pulmonary Disease (COPD) is the most common respiratory disease in clinic. Traditional Chinese medicine (TCM) lung rehabilitation has gradually been valued in the field of prevention and treatment of COPD. Acupuncture, as an important part of TCM lung rehabilitation, is carried out in clinical extensively and application. It has the advantage of improving the symptoms and quality of life of COPD cases, but the evidence-based proof is limited.

**Methods:**

Eight databases will be systematically retrieved from their inceptions to December 2021. This study will include randomized controlled trials of acupuncture in the treatment of COPD with stable stage. The main outcome will include clinical effective rate, TCM symptom score, quality of life, dyspnea, exercise capacity, lung function, frequency of acute exacerbation, adverse events. Literature screening, quality evaluation and information extraction will be completed by two independent researchers. Revman 5.3 software will be used to perform meta-analysis. Assessment of multiple systematic reviews-2 tool will be used for evaluating the methodological quality of systematic review (SR), grading of recommendations assessment will for rating the quality of evidence, and consolidated standards of reporting trials and Standards for Reporting Interventions in Clinical Trials of Acupuncture tool will for assessing the reporting quality of randomized controlled trials.

**Results:**

The findings of this SR will be presented at relevant conferences and submitted for peer-review publication.

**Conclusions:**

The findings of this SR will provide up-to-date summary proof for evaluating the effectiveness and safety of acupuncture for COPD.

## 1 Introduction

Chronic obstructive pulmonary disease (COPD) is a chronic airway disease characterized by persistent respiratory symptoms and airflow restriction.^[1]^ It is usually caused by airway and /or alveolar abnormalities caused by long-term exposure to toxic particles or gases.^[1]^ It is the most common chronic airway disease in clinic and an important cause of death.^[2]^ Chronic cough, expectoration and shortness of breath after activities are the main clinical manifestations of COPD, which seriously affects the quality of life (QoL) of patients and brings a heavy economic burden to their families and society.^[3]^

According to the report of the World Trade Organization, the number of patients with COPD reached 251 million in 2016, resulting in 3 million deaths, accounting for 5.3% of the annual death toll and it is expected to reach 6.7% in 2060.^[4]^ A research report shows that in 2018, the prevalence of COPD in adults aged 20 and over in China was 8.6%, and aged 40 and over was as high as 13.7% and it is estimated that there are nearly 100 million patients in China.^[5]^ At present, the mainstream belief among scientists holds that COPD is the result of the interaction of a variety of environmental and personal factors, such as smoking, air pollution, occupational dust exposure, low socio-economic status, gender, age, genetic polymorphism, etc. among them, smoking is considered to be the main environmental factor inducing the disease.^[6]^

In recent years, lung rehabilitation has been paid more and more attention in the field of COPD prevention and treatment. In 2019, the Global Initiative for Chronic Obstructive Lung Disease(GOLD)defined the important position of lung rehabilitation in the comprehensive management of COPD in remission stage.^[7]^ Lung rehabilitation plays a significant role in improving patients’ clinical symptoms, improving QoL and reducing complications.^[8]^ As an important part of lung rehabilitation, Traditional Chinese Medicine(TCM) lung rehabilitation is widely carried out in clinic which include acupuncture, acupoint application, moxibustion and Tai Chi, etc.^[9]^ Holistic view and syndrome differentiation and treatment are the core concepts of TCM. TCM lung rehabilitation is a comprehensive rehabilitation measure to promote the functional recovery of patients by adopting TCM rehabilitation technology for lung diseases under the guidance of this core concept and combined with modern rehabilitation technology.^[10]^

As an important part of external treatment of TCM, acupuncture is widely used in the prevention and treatment of a variety of diseases.^[11]^ Acupuncture is to stimulate the corresponding acupoints of the human body, inspire the meridians Qi of the human body, regulate the functions of the human viscera and improve the internal environment, so as to achieve the purpose of treating and preventing diseases. It has the advantages of simplicity, safety, economy and wide application range.^[12]^ The pathogenesis of COPD is closely related to inflammatory response, oxidative stress and protease-anti protease imbalance. At present, there are relatively few studies on the mechanism of acupuncture in the treatment of COPD, and its mechanism may be closely related to the inhibition of inflammation and immune regulation.^[13]^

At present, we have retrieved two systematic reviews of acupuncture in the treatment of stable COPD. One systematic review (SR)^[14]^ have synthesized 9 randomized controlled trials (RCTs) and the results showed that acupuncture can increase pulmonary ventilation function (FEV1%, FVC), improve exercise tolerance (6MWD) and improve QoL (SGRQ) in patients with COPD, but it still needs to be further confirmed by large-scale high-quality randomized controlled trials(RCTs). The other one^[15]^ pooled 19 RCTs and the results indicated that acupuncture tend to ameliorate the functional effects (6MWD) and QoL (SGRQ) of COPD suffers, however, high-quality RCTs are still needed to demonstrate the effectiveness and safety of acupuncture COPD therapy.

In recent years, with the wide application of TCM lung rehabilitation in COPD cases, the number of trails assessing the effectiveness and safety of acupuncture alone or combined with other interventions in the treatment of COPD in stable stage has increased year by year.^[16]^ One study used acupuncture combined with Chinese medicine to treat 60 patients with stable COPD based on routine treatment of western medicine, and the result suggested that compared with the control group, it can effectively improve lung function, reduce clinical manifestations and improve the QoL.^[17]^ One research adopted acupuncture combined with Chinese medicine to treat patients with stable COPD and the results showed that compared with the control group, it can significantly improve the ventilation function, immune function, and clinical effective rate.^[18]^

The purpose of this SR is to summarized these RCTs latest published and provide up to date evidence-based proof for acupuncture in the treatment of COPD in remission stage.

## 2 Methods and analysis

### 2.1 Objective

This SR and meta-analysis will comprehensively summarize the latest published clinical evidence of acupuncture alone or combined with other interventions in the treatment of COPD in remission stage, so as to systematically evaluate its effectiveness and safety. Compared with published SRs, this study will pay more attention to the methodological quality of clinical research in order to obtain reliable evidence.

### 2.2 Study registration

This protocol has been registered on International Platform of Registered Systematic Review and Meta-analysis Protocols (INPLASY no. 2021120081, https://inplasy.com/) and we will report this study according to Preferred reporting items for systematic review and meta-analysis protocols 2015 (PRISMA-P 2015).^[19]^ The SR and meta-analysis will be carried out in accordance with the protocol formulated in advance, and reported according to the SR report update item PRISMA 2020.^[20]^ Any modification during the implementation will be described in the SR.

### 2.3 Inclusion and exclusion criteria

#### 2.3.1 Type of study

This study will only include RCTs of acupuncture alone or combined with other interventions in the treatment of stable COPD. We will only focus on the language of clinical studies in Chinese and English. Since the RCT with acupuncture as the main intervention has certain difficulty in the implementation of blind method, we will not emphasize whether blind method is adopted in the execution of clinical research. Observational studies, animal experiments, literature review, case reports, repeatedly published articles, experience summary, protocol, etc. will be excluded.

#### 2.3.2 Type of participants

Patients should be diagnosed as stable COPD by guidelines or expert consensus with clear diagnostic criteria. The patient's age should be greater than 18 years old, and there will be no restrictions on the patient's gender, race, course of disease and severity. Patients with other respiratory diseases such as asthma, bronchiectasis, pulmonary fibrosis, lung cancer and severe liver, kidney and heart diseases will be excluded. Patients in acute exacerbation of COPD will not meet the inclusion criteria.

#### 2.3.3 Type of interventions

The intervention measures in control group can include sham acupuncture, routine treatment of western medicine, Chinese medicine, acupoint application, moxibustion and so on. The intervention measures in treatment group should be combined with acupuncture or acupuncture plus other intervention measures on the basis of control group. Acupuncture mainly includes body acupuncture, scalp acupuncture, eye acupuncture, ear acupuncture, etc. There will be no restrictions on acupoint selection, time and course of treatment.

#### 2.3.4 Type of outcome measurements

The outcomes we focus on are as follow:^[21]^ Clinical effective rate; TCM symptom score; quality of life (COPD assessment test, St. George respiratory questionnaire, chronic respiratory questionnaire, etc); dyspnea (borg scale, visual analog dyspnea scale, modified British medical research council respiratory diffificulty questionnaire, etc); exercise capacity (6-minute walking test, shuttle walking test, etc); lung function (FEV1, FVC, FEV1/FVC, TLC, RV, etc); frequency of acute exacerbation; adverse effects.

### 2.4 Search strategy

We will retrieve the following 8 databases: PubMed, EMBASE, Cochrane Central Register of Controlled Trials, Web of Science, China National Knowledge Infrastructure, WangFang Database, Chinese Science and Technology Periodical Database, SinoMed. The retrieval time is from the establishment of each database to December 2021. The method of subject words combined with free words for retrieval will be applied, and the retrieval formula will be formulated and adjusted according to the characteristics of each database. The language of the literature is limited to Chinese and English. We will also retrieve clinical trial registration platforms such as Clinical Trials.gov trials registry, Chinese Clinical Trial Registry, etc. to track ongoing or completed clinical studies. If possible, we will try to contact the researchers to obtain the required research data. Grey literature and references of published SRs will also be searched to avoid omissions. We will also manually search the catalogue of the following journals to avoid the omission of high-quality RCTs that meet the standards: Chinese Acupuncture & Moxibustion, Acupuncture Research, Acupuncture in Medicine, World Journal of Acupuncture Moxibustion, Journal of Acupuncture and Meridian Studies, Medical Acupuncture, Journal of Acupuncture and Tuina Science, Acupuncture & Electro-theraputics Research.

### 2.5 Search strategy in Pubmed

(“Pulmonary Disease,Chronic Obstructive”[Mesh Terms]OR“Chronic Obstructive Pulmonary Diseases”[Title/Abstract]OR“COPD”[Title/Abstract]OR“Chronic Airflow Obstructive”[Title/Abstract]OR“Chronic Obstructive Lung Disease”[Title/Abstract]) AND(acupuncture[MeSH Terms]OR electroacupuncture[MeSH Terms]OR ear acupuncture[Title] OR auricular acupuncture[Title]OR ear needling[Title]OR scalp acupuncture[Title]OR head needle[Title]OR head acupuncture[Title]OR pyonex[Title] OR plum-blossom needle[Title]OR percussopunctator[Title]OR eye needle[Title]OR eye acupuncture[Title])AND(randomized controlled trial[MeSH Terms])

### 2.6 Studies selection

**Figure 1:**
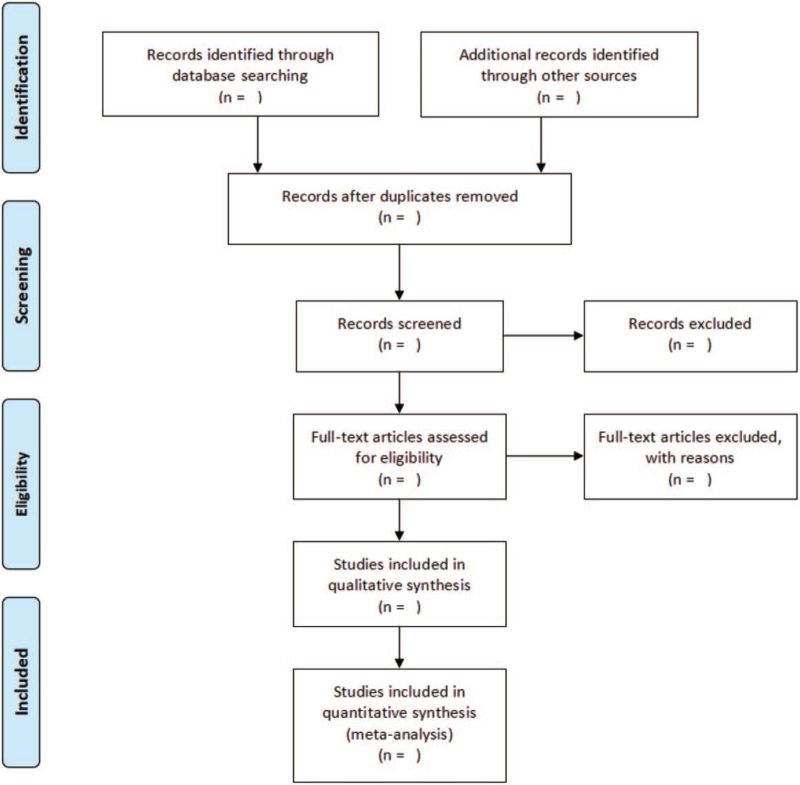
Flow diagram of study selection process legend: details of the selection process will be presented in the PRISMA flow chart.

All retrieved literature will be input into Note Express software (version 3.0) to eliminate duplicate documents. Firstly, two researchers will conduct preliminary screening by reading the title and abstract according to the pre-determined inclusion and exclusion criteria. The retained articles will be further filtered by reading the full text after downloading. Any differences arising in this process will be decided through consultation or by a third senior researcher. All excluded articles will provide appropriate reasons for exclusion. Details of the selection process will be presented in the PRISMA flow chart (Fig. 1).

### 2.7 Data extraction

Two investigators will use a pre-designed extraction form to extract the following information from the included RCTs: the first author, publication time, sample size, intervention measures, control measures, acupuncture points, diagnostic criteria, outcomes, funding. If there is missing or unclear information in RCTs, we will try to contact the author to obtain the corresponding information. Differences arising in this process will be decided through consultation or by a third senior researcher.

### 2.8 Risk of bias and literature quality assessment

Cochrane risk of bias assessment tool^[22]^ will be used to assess the risk of bias in the included RCTs. Based on the risk of bias assessment items, two researchers will evaluate from six aspects, namely “random sequence generation” “allocation concealment” “blinding methods” “incomplete outcome data” “selective outcome reporting” “other bias”. For each item, the evaluator will make judgments of “low-risk bias”, “high-risk bias” or “unclear”. Finally, the evaluation results of each study will be summarized and displayed in the form of charts.

Modified Jadad scale^[23]^ will be used to evaluate the quality of RCTs. The two researchers will assess the included RCTs from four aspects, namely “randomization”, “concealment of allocation”, “double blinding”, “withdrawals and dropouts”. According to the scoring principle, 1-3 points will be considered as low-quality literature, and 4-7 points as high-quality literature.

Differences arising in this process will be decided through consultation or by a third senior researcher.

### 2.9 Strategy for data synthesis

In this SR, Revman 5.3 software will be used to perform meta-analysis. The *Q*-test and *I*^2^ values will be applied to measure the inter-study heterogeneity. When the *P* value of *Q* test >.1 and *I*^2^ < 50%, a fixed effects model will be applied; When *P* value of *Q* test <.1 and *I*^2^ > 50%, we will try to explore the causes of heterogeneity. If the heterogeneity is still significant, random effect model will be used for analysis. Binary variables were expressed using the odds ratio with 95% confidence interval (CI) and continuous variables by the standardized mean difference with 95% CI.

### 2.10 Subgroup analysis

The clinical heterogeneity and / or methodological heterogeneity of the included RCTs are the main reasons for the statistically significant heterogeneity. Subgroup analysis will be used to explore potential factors causing significant heterogeneity, such as intervention measures, control measures, study quality, sample size, course of treatment, risk of bias, etc.

### 2.11 Sensitivity analysis

The combination of different levels of risk of bias and quality of studies may affect the overall effect. We will try to eliminate the low-quality RCT according to the assessments results of risk of bias and methodological quality to confirm the stability of conclusion. If the overall quality of RCTs contained under the outcome are similar, the method of excluding RCT one by one will be used for sensitivity analysis.

### 2.12 Publication biases

Articles with positive results may be easier to publish, thus exaggerating the clinical effects of interventions. Therefore, it is necessary to evaluate the publication bias of included studies. If the number of RCTs included is more than 10, the inverted funnel chart will be used to evaluate the potential publication bias. If the inverted funnel diagram is approximately symmetrical, there is less possibility of publication bias, otherwise, publication bias may exist.

### 2.13 Rating the confidence in estimates of the effect

Grading of Recommendations Assessment, Development, and Evaluation (GRADE) offers a transparent and structured process for developing and presenting summaries of evidence, including its quality, for systematic reviews.^[24]^ The GRADE system will be used to assess the quality of evidence. Two pre-trained researchers will rate the quality of evidence from 5 aspects (inconsistency, imprecision, limitations/risk of bias, publication bias and indirectness) according to specific criteria. The overall quality of evidence may be rated as “high” “moderate” “low” or “very low.” Differences arising in this process will be decided through consultation or by a third senior researcher.

### 2.14 Assessment of methodological quality

Assessment of multiple systematic reviews-2 (AMSTAR-2)^[25]^ is the most commonly used tool for evaluating the methodological quality of SR and meta-analysis. According to AMSTAR-2 guidance document, the overall methodological quality of SR may be rated as “high”, “moderate”, “low,” and “critically low”. Online AMSTAR-2 checklist (http://www.amstar.ca/Amstar_Checklist.php) will be applied to calculate and complete the scores in this research. This process will be completed by two researchers, and any divergence will be adjudicated through consultation or by a third senior researcher.

### 2.15 Quality assessment of RCT included

Consolidated standards of reporting trials (CONSORT)^[26]^ is a scale designed to improve the transparency and reporting quality of RCTs. Standards for Reporting Interventions in Clinical Trials of Acupuncture (STRICTA)^[27]^ aims to improve the integrity and transparency of intervention reports in acupuncture controlled trials, so that other researchers or clinical practitioners can clearly explain and easily repeat such trials. CONSORT and STRICTA tools will be used to evaluate the reporting quality of the RCTs included. Differences during this process will be decided through negotiation or by the third senior researcher.

### 2.16 Ethics and dissemination

This SR will not require ethical approval because no data are used in our study that are linked to individual patient data. Besides, findings will be disseminated through peer-review publications.

### 2.17 Strengths and limitations of this study

1. This study will be up to date SR and meta-analysis to summarize the latest evidence of RCTs of acupuncture in stable COPD therapy;
2. AMSTAR-2 tool will be applied to assess the methodological quality of this SR,so as to ensure the transparency of SR and meta-analysis and the reliability of conclusions;
3. GRADE system will be used to rate the quality of proof to clarify the recommended strength of evidence;
4. CONSORT and STRICTA scales will be used to assess the reporting quality of RCTs included in the SR;
5. This SR will only include Chinese and English literature,which may lead to omission.

## 3 Discussion

Chronic obstructive pulmonary disease ranks the third cause of death in China, and its prevalence and mortality are on the rise.^[28]^ TCM lung rehabilitation plays an significant role in improving patients’ symptoms and QoL. The Clinical Application Guidelines of Traditional Chinese Medicine Lung Rehabilitation for Chronic Obstructive Pulmonary Disease^[29]^ was officially published in 2021, and acupuncture was included as an important and effective intervention. Acupuncture combined with conventional western medicine is recommended in the guideline to enhance the QoL, improve lung function and reduce adverse reactions, nevertheless, the evidence is limited and the level is not high.^[29]^ The purpose of this SR is to comprehensively collect and systematically assess the RCTs of acupuncture in the treatment of COPD in remission stage, which in order to provide latest and reliable evidence-based proof. Considering the quality of differences in RCTs included, Cochrane risk of bias assessment tool and modified Jadad scale will be adopted to appraise them, and we will fully take into account the potential impact of literature quality differences on the results, but it may still be difficult to avoid. Therefore, large sample, multi-center and high-quality RCTs are suggested to design and implement in the future and report them based on consort and stricta scale, and report them based on CONSORT and STRICTA scale, in order to obtain high-quality evidence-based proof to guide clinical practice.

## Author contributions

**Conceptualization:** Tianyi You, Tie-Ming Ma.

**Funding acquisition:** Ji-Quan Li, Tie-Ming Ma.

**Investigation:** Tianyi You, Hao-Yang Zhang, Tie-Ming Ma.

**Methodology:** Tianyi You.

**Supervision:** Tie-Ming Ma.

**Writing – original draft:** Tianyi You.

**Writing – review & editing:** Tianyi You, Hao-Yang Zhang, Ji-Quan Li, Tie-Ming Ma.
